# Torque Teno Virus and Hepatitis C Virus Co-Infection in Iranian Pediatric Thalassemia Patients

**DOI:** 10.5505/tjh.2012.20280

**Published:** 2013-05-15

**Authors:** Samin Alavi, Ali Kord Valeshabad, Zohreh Sharifi, Kazem Nourbakhsh, Mohammad Taghi Arzanian, Masoumeh Navidinia, Siamak Mehdizadeh Seraj

**Affiliations:** 1 Mofid Children’s Hospital, , Pediatric Hematology-Oncology Department, and Pediatric Infectious Research Center, Shahid Beheshti University of Medical Sciences, Tehran, Iran; 2 Students’ Scientific Research Center (SSRC), Tehran University of Medical Sciences, Tehran, Iran; 3 Iranian Blood and Transfusion Organization, Tehran, Iran; 4 Pediatric Infectious Research Center, Shahid Beheshti University of Medical Sciences, Tehran, Iran

**Keywords:** Thalassemia, Torque teno virus, Hepatitis C virus

## Abstract

**Objective:** Torque teno virus (TTV) infects patients at risk for parenteral exposure and chronic blood transfusion, such as those with β-thalassemic. This study aimed to assess the prevalence of TTV infection and co-infection of TTV and hepatitis C virus (HCV) in pediatric thalassemia patients receiving chronic blood transfusion.

**Material and Methods: **The study included 90 pediatric thalassemia patients receiving chronic blood transfusion that presented to the Mofid Children’s Hospital, Tehran, Iran. The control group included 90 healthy volunteer children. Serum TTV DNA detection via semi-nested PCR and HCV Ab were performed in all the participants. Demographic characteristics and clinical data were collected from each participant for statistical analysis.

**Results:** In all, 64.4% of the patients had TTV infection, versus 24.4% of the controls (P < 0.01). The thalassemia patients had a greater probability of having TTV and HCV infections than the controls, with a common OR of 5.60 (95% CI: 2.94-10.69) and 2.15 (95% CI: 1.83-2.50), respectively. In total, 17.2% (10/58) of the patients that were TTV positive were also HCV positive, whereas 6.3% (2/32) of the TTV-negative patients were anti-HCV antibody (Ab) positive (P = 0.14).

**Conclusion:** The prevalence of TTV and HCV infection was higher in the Iranian thalassemia patients on chronic transfusion therapy than in the controls. The high prevalence of TTV in pediatric thalassemia patients on chromic transfusion therapy may indicate the superiority of the parenteral route compared to other routs of TTV transmission.

## Abstract (tr)

**Amaç:** Torque teno virüsü (TTV) beta talasemik hastalar gibi kronik kan transfüzyonu ve parenteral yoldan maruz kalma açısından risk altındaki hastaları enfekte eder. Bu çalışma kronik kan transfüzyonu alan pediatrik talasemi hastalarında TTV enfeksiyonu ve TTV ile hepatit C virüsü (HCV) koenfeksiyonunun prevalansını değerlendirmeyi hedeflemiştir.

**Gereç ve Yöntemler:** Çalışmaya İran’da Tahran’da Mofid Çocuk Hastanesine gelen ve kronik kan transfüzyonu alan 90 pediatrik talasemi hastası dahil edilmiştir. Kontrol grubu 90 sağlıklı gönüllü çocuğu içermiştir. Tüm katılımcılarda semi-nested PCR ve HCV Ab yoluyla serum TTV DNA’sı saptaması yapılmıştır. Her katılımcıdan istatistiksel analiz için demografik özellikler ve klinik veriler toplanmıştır.

**Bulgular:** Genel olarak hastaların %64,4’ünde ve kontrol grubunun %24,4’ünde TTV enfeksiyonu bulunmuştur (P < 0,01). Talasemi hastalarında kontrol grubuna göre TTV ve HCV enfeksiyonu bulunması olasılığı olması daha yüksek olmuştur ve ortak risk oranı sırasıyla 5,60 (%95 GA: 2,94-10,69) ve 2,15 (%95 GA: 1,83-2,50) bulunmuştur. Toplam olarak TTV pozitif hastaların %17,2’si (10/58) HCV pozitifken TTV negatif hastaların %6,3’ü (2/32) anti HCV antikoru (Ab) pozitiftir (P = 0,14).

**Sonuç:** TTV ve HCV enfeksiyonu prevalansı kronik transfüzyon tedavisi alan İranlı talasemi hastalarında kontrollerden daha yüksek bulunmuştur. Kronik transfüzyon tedavisi alan pediatrik talasemi hastalarında yüksek TTV prevalansı parenteral yolun diğer TTV bulaşması yollarına göre daha sık kullanılmasına işaret edebilir.

## INTRODUCTION

Patients with β-thalassemia major are prone to transfusion- related hepatitis because of chronic dependency on blood transfusion, and associated transfusion-related iron overload and transmission of viruses. Although the implementation of screening for hepatitis B and C virus (HBV and HCV) nuclear acid and antibodies has substantially reduced the incidence of transfusion-related hepatitis, a considrable proportion of thalassemia patients still have elevated liver enzymes of unknown origin [1,2]. In 1997 a novel DNA virus—transfusion-transmitted virus (TTV)— was cloned by a Japanese team from 3 patients with posttransfusion non-A-G hepatitis [3]. This non-enveloped single-stranded DNA virus was renamed Torque teno virus, a species of the genus Anelloviridae in an unassigned family that is most closely related to Circoviridae [4,5]. TTV infects patients at risk for parenteral exposure, such as those with thalassemia, hemophilia, and liver disease [6,7,8,9], but it can be found in other body fluids and secretions, including saliva, semen, stool, breast milk, and tears [10]. Recently, its routes of vertical and sexual transmission have been reported [11]. 

Primary epidemiological studies from the United Kingdom and Japan reported that TTV DNA was detectable in 25%-45% of patients with chronic or fulminant hepatitis of unknown origin, in 27%-68% of hemophiliacs, and in 1.9%-12% of healthy blood donors [6,7,12]. The prevalence of TTV among thalassemia patients was reported to vary from 50% to 100% in different studies and appears to be dependent upon diagnostic techniques, study sample size, and geographic distribution [13]. Even its prevalence among the general population has been reported to range from 1% in the North America to as high as 54% in Turkey [14,15]. Despite the high prevalence of this virus among thalassemia patients, its potential role in cryptogenic or post-transfusion hepatitis is unclear, as most TTV-positive patients remain asymptomatic and those that progress to chronic liver disease are invariably co-infected with other hepatitis viruses [8]. There are a limited number of studies in Iran about the prevalence of TTV and its clinical importance in thalassemia patients. It remains unknown if TTV infection in thalassemia patients increases the incidence of co-infection with other hepatitis viruses such as hepatitis C As such, the present study aimed to determine the prevalence of TTV infection and co-infection of TTV and HCV in pediatric thalassemia patients on chronic transfusion therapy in Iran.

## MATERIALS AND METHODS

This case-control study included all pediatric thalassemia patients on chronic transfusion therapy treated at the Mofid Children’s Hospital, Tehran, Iran, and a control group of healthy volunteer children. The patients included had presented to the orthopedic outpatient clinic due to minor injuries and without a history of prior transfusion, hepatitis, surgery, parenteral treatment, or systemic diseases. All the patients were systematically examined by pediatrics residents. Informed consent was obtained from the patients or their parents if aged <18 years. Primary screening tests, including human immunodeficiency virus (HIV), human T-cell leukemia, and hepatitis B and C were performed in all the study participants and those with any positive result were excluded. TTV DNA and anti-HCV antibody (anti-HCV Ab) testing were performed in all the participants to determine their prevalence rates. 

Anti-HCV status was determined using 2[u]nd[/u]- and 3[u]rd[/u] generation assays (Ortho Diagnostic Systems, Raritan, NJ). Hepatitis B surface antigen (HBsAg) status was determined via enzyme immunoassay (EIA) (Murex, Dartford, UK; and Abbott Laboratories, Chicago, IL). HCV RNA was measured via reverse transcriptase polymerase chain reaction (RT-PCR), as previously described [16]. Demographic characteristics and clinical data, including age, gender, transfusion duration, and anti-HCV Ab and TTV DNA test results were collected from each participant for statistical analysis. The study protocol was approved by the Shahid Beheshti University of Medical Sciences Ethics Committee. 

**Isolation and determination of TTV DNA via PCR **

Semi-nested PCR was used to detect serum TTV DNA. Specifically, serum DNA purified from an equivalent of 7 μL of serum was amplified according to the following PCR protocol in a 9600 thermal cycler (Perkin-Elmer, Emeryville, CA): 1 cycle at 95 °C for 9 min; 35 cycles at 94 °C for 30 s, 58 °C for 30 s, and 72 °C for 45 s; 1 cycle at 72 °C for 7 min. The reaction conditions were as follows: 30 pmol of each primer (sense NG059 5’-ACA GAC AGA GGA GAA GGC AAC ATG-3’, antisense NG063 5’-CTG GCA TTT TAC CAT TTC CAA AGT-3’) and 2.5 U of AmpliTaq Gold DNA polymerase (Perkin-Elmer, Norwalk, CT) in a 50-μL reaction volume. Under the same conditions the second round of PCR was performed using a semi-nested primer set (sense NG061 5’ GGC AAC ATG TTA TGGATA GAC TGG 3’, antisense NG063) for 5 μL of the amplification product. In each PCR assay 2 positive and negative controls were included. Then, the PCR products were analyzed via 2% agarose gel electrophoresis with ethidium bromide staining. Separate assays were performed for all positive samples of TTV DNA and the sequences of the PCR products were confirmed via automated sequencing on an ABI 373 sequencer (Perkin-Elmer, Foster City, CA). NG hemi-nested PCR, using primers NG059, NG061, and NG063 [6], has been used in many studies, but has been shown to have suboptimal sensitivity [17,18,19]. 

**Statistical analysis**

Results are reported as mean ± standard deviation (SD) for quantitative variables and percentages for categorical variables. The 2 groups were compared using Student’s t-test for continuous variables and the chi-square test (or Fisher’s exact test if required) for categorical variables. Statistical significance was based on two sided tests evaluated at the P = 0.05 level of significance. All statistical analyses were performed using SPSS v.13.0 (SPSS Inc, Chicago, IL, USA) for Windows.

## RESULTS

In total, 121 pediatric thalassemia patients and 134 healthy controls were evaluated, and 31 of the thalassemia patients and 44 of the controls were excluded based on the study’s exclusion criteria. TTV and HCV prevalence rates were determined in 90 thalassemia patients (46 boys and 44 girls) with the mean age of 14.1 ± 5.8 years and 90 healthy controls (44 boys and 46 girls) with the mean age of 13.3 ± 6.4 years. The patient and control groups did not significantly differ in mean age (P = 0.61) or gender (P = 0.48). In all, 64.4% of the patients were TTV seropositive, versus 24.4% of the controls (P < 0.001). The thalassemia patients had a greater probability of TTV and HCV seropositivity than the controls, with a common OR of 5.60 (95% CI: 2.94-10.69, P = 0.001) and 2.15 (95% CI: 1.83- 2.50, P = 0.001), respectively. 

In the patient group a significant difference was not observed in mean age, transfusion duration, or TTV and HCV seropositivity between the boys and girls (P > 0.05). In the control group mean age was statistically higher in the girls than in the boys (14.3 ± 6.8 years versus 11.4±4.7 years (P=0.04), whereas TTV and HCV seropositivity did not differ between genders (P > 0.05). Table 1 shows the demographic and some clinical data for the 2 groups. 

In all, 58 of the patients (31 boys and 27 girls) were TTV positive and 32 (15 boys and 17 girls) were TTV negative. Age, gender, and transfusion duration did not differ significantly between the TTV-positive and TTV-negative thalassemia patients (P > 0.05). In all, 17.2% (10/58) of the TTV-positive patients had simultaneous HCV seropositivity, whereas 6.3% (2/32) of the TTV-negative patients were positive for anti-HCV Ab; the difference was not statistically significant (P = 0.14) (Table 2). Among the TTV-positive thalassemia patients, there was not a significant difference in mean age, transfusion duration, or anti-HCV Ab status between the boys and girls (P > 0.05). Similarly, among the TTV-negative thalassemia patients, mean age, transfusion duration, and anti-HCV Ab status did not differ significantly between the boys and girls (P>0.05) (Table 2). Table 2 shows the demographic and some clinical data for the TTV-positive and TTV-negative thalassemia patients.

## DISCUSSION

The potential role and pathogenesis of TTV infection in post-transfusion hepatitis has yet to be established [20,21]. Although TTV was initially considered a new transfusiontransmitted virus in patients with acute and chronic non- A-G hepatitis, subsequent studies raised doubts about the hypothesis that TTV infection leads to clinical manifestation in all infected patients [15]. In the present study pediatric thalassemia patients had a greater probability of TTV and HCV seropositivity than the controls, with a common OR of 5.60 (95% CI: 2.94-10.69, P = 0.001) and 2.15 (95% CI: 1.83-2.50, P = 0.001), respectively. As previously reported [22,23], this finding may indicate the superiority of the parenteral route compared to other routes of TTV transmission. In all, 64.4% of the present study’s pediatric thalassemia patients had TTV, whereas only 24.4% of controls, which did not have a history of blood transfusion, hepatitis, parenteral treatment, or any known diseases, were TTV positive. The infected controls might have been infected via non-parenteral routes, such as saliva, semen, stool, breast milk, or tears [10]. 

Zandieh et al. reported that 57.2% of Iranian thalassemia patients and 20% of healthy controls were TTV positive [24]. The reported prevalence of TTV infection varies by study due to differences in diagnostic techniques, study sample size, and geographic distribution [13]; even sequencing of TTV clones from thalassemia patients showed that 1 patient had multiple TTV variants [13]. Ozyürek et al. reported that 63% of Turkish thalassemia patients had the virus [15]; additionally, 73% and 69% of Italian pediatric and adult thalassemia patients, respectively, had the virus [25,26], and 73.4% of thalassemia patients in Taiwan had the virus [23]. Such variation in the prevalence of TTV infection has been noted in the general population of different countries. Epidemiological studies reported that the prevalence of TTV infection is 1% in North America [20], 10% in Europe [27], 10%-62% in South America [10], 22% in Italy, 34% in Japan [14], and 54% in Turkey [15]. 

Age, gender, and transfusion duration did not differ significantly between the present study’s TTV-positive and TTV-negative thalassemia patients (P>0.05). In contrast, previous evaluation of TTV infection in 250 thalassemia patients in Ahwaz (a province in southern Iran) showed that there was a significant correlation between TTV infection, and age and history of blood transfusion [24]. The present study included pediatric thalassemia patients in Tehran and that other study included patients from southern Iran (Ahwaz); however, in addition to the difference in geographic location of the 2 study populations, they were ethnically different. It has been reported that the distribution of TTV infection varies with geographic region and country, which might account for the differences between the 2 studies’ findings [10,14,15,20,23,27]. 

The present study has some limitations. The presented data and results were based on TTV DNA amplification via PCR using only 1 set of primer. It is possible that other variants of TTV DNA existed in our samples that were not detectable using this set. Samples found to be positive via NG hemi-nested PCR may subsequently undergo restriction fragment length polymorphism analysis for genotype identification, but this was not applied in the present study. The clinical impacts of this virus on liver function enzymes and histopathologic changes were not assessed in all the patients and were not used in the analyses. Thus, we plan to design studies in the future to assess the clinical importance and features of TTV in thalassemia patients. 

**Conclusion**

The prevalence of TTV and HCV was higher in the pediatric thalassemia patients on chronic transfusion therapy than in the healthy controls. The high prevalence of TTV in this group of patients may indicate the superiority of the parenteral route compared to other routes of TTV transmission. 

**Conflict of Interest Statement **

The authors of this paper have no conflicts of interest, including specific financial interests, relationships, and/ or affiliations relevant to the subject matter or materials included.

## Figures and Tables

**Table 1:**
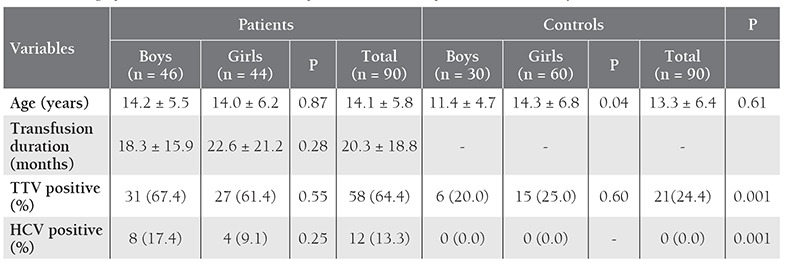
Demographic and clinical data for the 90 pediatric thalassemia patients and 90 healthy controls.

**Table 2:**
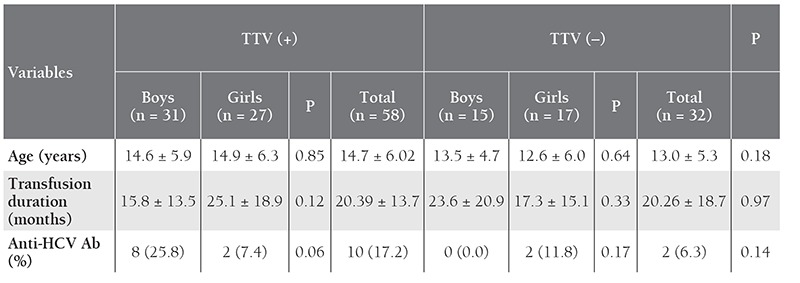
Demographic and clinical data for the TTV-positive and TTV-negative thalassemia patients.
